# Adverse childhood experiences and adult cardiometabolic risk factors and disease outcomes: Cross-sectional, population-based study of adults in rural Uganda

**DOI:** 10.7189/jogh.11.04035

**Published:** 2021-07-24

**Authors:** Andrew Wooyoung Kim, Bernard Kakuhikire, Charles Baguma, Crystal M North, Emily N Satinsky, Jessica M Perkins, Patience Ayebare, Allen Kiconco, Elizabeth B Namara, David R Bangsberg, Mark J Siedner, Alexander C Tsai

**Affiliations:** 1 SAMRC/Wits Developmental Pathways for Health Research Unit, Faculty of Health Sciences, University of the Witwatersrand, Johannesburg, South Africa; 2 Center for Global Health, Massachusetts General Hospital, Boston, Massachusetts, USA; 3 Mbarara University of Science and Technology, Mbarara, Uganda; 4 Division of Pulmonary and Critical Care, Massachusetts General Hospital, Boston, Massachusetts, USA; 5 Mongan Institute, Massachusetts General Hospital, Boston, Massachusetts, USA; 6 Harvard Medical School, Boston, Massachusetts, USA; 7 Peabody College, Vanderbilt University, Nashville, Tennessee, USA; 8 Oregon Health and Science University – Portland State University School of Public Health, Portland, Oregon, USA; 9 Medical Practice Evaluation Center and Division of Infectious Diseases, Massachusetts General Hospital, Boston, MA, USA

## Abstract

**Background:**

Cardiovascular diseases (CVD) pose a major threat to public health in sub-Saharan African communities, where the burden of these classes of illnesses is expected to double by 2030. Growing research suggests that past developmental experiences and early life conditions may also elevate CVD risk throughout the life course. Greater childhood stress and adversity are consistently associated with a range of adult CVDs and associated risk factors, yet little research exists on the long-term effects of early life stress on adult physical health outcomes, especially CVD risk, in sub-Saharan African contexts. This study aims to evaluate the associations between adverse childhood experiences and adult cardiometabolic risk factors and health outcomes in a population-based study of adults living in Mbarara, a rural region of southwestern Uganda.

**Methods:**

Data come from an ongoing, whole-population social network cohort study of adults living in the eight villages of Nyakabare Parish, Mbarara. A modified version of the Adverse Childhood Experiences-International Questionnaire (ACEs) assessed past exposure to physical, emotional, and sexual adversity. Participants also took part in a health fair where medical histories on cardiometabolic risk factors and cardiovascular diseases were gathered. Multiple logistic regression models estimated the associations between ACEs and cardiometabolic risk factors and health outcomes.

**Results:**

Data were available on 545 adults. The average number of ACEs was 4.9 out of a possible 16. The cumulative number of ACEs were associated with having a history of heart attack and/or heart failure (adjusted odds ratio (AOR) = 1.11, 95% confidence interval (CI) = 0.999-1.234, *P* = 0.051), but the estimated association was not statistically significant. ACEs did not have statistically significant associations with any others measures of adult cardiometabolic risk and CVD.

**Conclusions:**

Adverse childhood experiences are not associated with a range of adult cardiometabolic risk factors and health outcomes in this sample of rural Ugandan adults. Further research in this sample is necessary to identify the pathways that may motivate these null relationship and possibly protect against adverse cardiometabolic and cardiovascular health outcomes.

Cardiovascular diseases (CVDs) are the leading cause of mortality globally, accounting for approximately 17.8 million deaths per year [1]. Increasing trajectories of CVD are especially concerning in sub-Saharan Africa, a region already facing a double burden of disease from non-communicable diseases (NCDs) and infectious diseases [2] and where the burden of CVD is expected to double by 2030 [3]. While dietary and lifestyle factors account for a major portion of overall CVD mortality and morbidity, growing research suggests that past developmental experiences and early life conditions between fetal development and late adolescence may also elevate CVD risk throughout the life course [4-6].

Research over the past two decades has found that childhood trauma and adversity, typically assessed by querying past experience with a variety of physical, emotional, and socioeconomic insults, are important long-term risk factors for adult CVD morbidity [7,8], mortality [9,10], and related cardiometabolic conditions [8] across diverse contexts. Greater childhood adversity has been consistently associated with a range of adult CVDs, including hypertension [6], stroke [11], myocardial infarction [12], and congenital heart disease [13], as well as major CVD risk factors [5]. Early adverse experiences are hypothesized to have durable impacts on CVD risk through the disruption of various psychological, biological, and socioeconomic pathways across child development. For instance, adults with greater self-reported childhood trauma face substantially higher risk for adult psychopathology, such as depression and posttraumatic stress disorder, occurring both independently and comorbid with CVD. These adult psychiatric conditions, along with early life stress, are commonly linked with alterations in stress physiological systems (eg, hypothalamic-pituitary-adrenal axis, sympathetic-adrenal-medullary-axis) that are also involved in the etiology of CVD, such as glucocorticoid dysregulation [14] and heightened systemic inflammation [15].

Despite the alarming rates of CVD burden in sub-Saharan Africa, most studies come from study populations in high-income countries [16,17]. In a recent meta-analysis of the global literature on childhood adversity and later-life health, Hughes et al. (2017) describe that “[l]ittle is known about how ACEs predict health outcomes in low-income, high-violence settings, where exposure to adversity is widespread across the life-course.” Very little evidence exists on the associations between childhood adversity and adult physical health outcomes, especially CVD risk, in sub-Saharan African contexts [17]. A number of studies from sub-Saharan Africa have shown associations between greater childhood adversity and worse mental health outcomes and self-reported health in adolescence and young adulthood [17-23], but few studies on adult health outcomes exist. To fill this gap in the literature, we estimated the associations between adverse childhood experiences and adult cardiometabolic risk factors and health outcomes in a population-based study of adults living in Mbarara, a rural region of southwestern Uganda.

## METHODS

### Study sample

This study was conducted in the eight villages of Nyakabare Parish, Mbarara, a rural region of southwestern Uganda. The study setting is representative of the larger rural conditions in Mbarara. Nyakabare Parish is geographically isolated, residents mostly engage in subsistence farming, petty trade, and migrant work for labor, and food and water insecurity are prevalent [24,25]. Data were drawn from the second wave of an ongoing, whole-population social network cohort study being conducted in the region [26]. The study includes all adults aged 18 years and above (and emancipated minors aged 16-17 years) who maintain stable primary residence in Nyakabare Parish and who can provide informed consent. Exclusions include people who cannot communicate meaningfully with research staff, for example, because of deafness, mutism, or aphasia; people with behavioral problems thought to represent psychosis, neurological damage, or acute intoxication; and people too cognitively impaired to provide informed consent. The overall response rate was 1630/1795 = 90.8%.

All participants provided written informed consent before completing all study procedures. Informed consent materials were explained verbally, and research assistants probed for comprehension and answered any questions. Study participants who could not provide a written signature were permitted to indicate consent with a thumbprint. All study procedures were approved by the Mbarara University of Science and Technology and Partners Healthcare human studies ethics committees. Per national guidelines, we received clearance to conduct the study from the Uganda National Council for Science and Technology and the Research Secretariat in the Office of the President in Uganda.

### Community survey data

In this study, a modified version of the Adverse Childhood Experiences-International Questionnaire was administered in the second wave (2016-18) to assess experiences of abuse, neglect, and household dysfunction during childhood [27]. The following 16 experiences were queried: 1) verbal abuse, 2) fear of harm, 3) being pushed/grabbed/slapped/hit by an object, 4) scarring from physical abuse, 5) sexually abused, 6) raped, 7) parental divorce, 8) pushed/grabbed/slapped/thrown an object at one’s mother, 9) kicked/bit/punched one’s mother, 10) threatened mother with weapon, 11) lived with an alcohol/drug abuser, 12) lived with adult with mental illness, 13) incarcerated family member, 14) experienced an entire day without food, 15) went to bed hungry, and 16) went to bed thirsty.

The survey also assessed a series of demographic characteristics, which included an asset inventory that queried the possessions of the following items: radio, lantern, bicycle, television, iron, motorbike, refrigerator, stove, car, and mobile phone; type of toilet facility; materials used to construct the household floors and walls; number of rooms in the home; number of plots of land owned; and number of livestock; and size of the household's rainwater harvesting tank, if any. We created asset index scores using principal component analysis, and households were categorized into quintiles of asset wealth (“wealth quintiles”) [28,29].

### Community health fair and biomarker data

During the study, all adults aged at least 18 years of age were invited to attend a multi-sited community-wide health fair [30]. Health fair staff administered study questionnaires, obtained blood samples, and conducted medical screenings. The following cardiometabolic risk factors and health outcomes were assessed: obesity status, defined by conventional waist circumference thresholds (≥102cm for men, ≥88cm for non-pregnant women) [31]; self-reported history of elevated blood pressure, diabetes, elevated cholesterol, heart attack/heart failure, stroke; hemoglobin A1c (%); and elevated blood pressure. A1c assessment was done at the time of blood collection using point-of-care Siemens Vantage A1c testing kits (Siemens Medical Solutions USA, Malvern, PA) [32]. Blood pressure was measured in a seated position using automated sphygmomanometers (Omron HEM 705 LP, Omron Healthcare, Inc., Bannockburn, IL), with elevated blood pressure defined as systolic blood pressure ≥140 mm Hg or diastolic blood pressure ≥90 mm Hg [33]. Body-mass index (BMI) was assessed by calculating the ratio of weight in kilograms divided by the square of height in meters.

### Statistical analysis

All analyses were conducted using Stata version 15.1 (Stata Corporation, College Station, TX, USA). We examined bivariate relationships to estimate the associations between adult health outcomes and ACEs and the other covariates. We then fitted multiple logistic regression models to the data to estimate the associations between ACEs and cardiometabolic risk factors and health outcomes. With the exception of psychological, household, and social factors that may confound the relationship between ACEs and adult cardiometabolic risk factors and health outcomes, only those variables that were statistically significant at the 0.1 level on bivariate analysis were included in the final multivariable regression models. The following variables were included in the final regression models: age, sex, household asset wealth, educational attainment, marital status, and BMI.

## RESULTS

Data from 545 adults who attended the community health fair and who participated in the community survey were included in this analysis. The mean age was 45.9 years (standard deviation [SD], 15.9), 62% were women, and most participants did not complete primary school (74.9%) (**Table 1**). Adults reported relatively high rates of clinically diagnosed hypertension (30.0%), and relatively lower levels of diabetes (2.5%), a past history of heart attack/heart failure (2.3%), and stroke (2.2%). Compared to those who participated in the community survey but who did not attend the community health fair, participants who attended the community health fair were more likely to be male, live with a partner, and report a negative history of diabetes.

**Table 1:** Socio-demographic and health characteristics of the sample

Variables	n = 545	%
**Demographics:**		
Gender (% female)	337	61.8
Age (years):		
18-25	40	7.3
26-35	122	22.4
36-45	130	23.9
46-55	118	21.7
56+	135	24.8
Educational attainment:		
No schooling	89	16.3
Some primary school	191	35.1
Completed primary school	126	23.1
Some secondary school or more	139	25.5
Living with partner:		
Yes	152	27.9
No	393	72.1
Self-reported outcomes:		
Diabetes	14	2.6
Heart attack/failure	31	5.7
High cholesterol	14	2.6
Hypertension	158	29.0
Stroke	11	2.0
*Measured outcomes:*		
Hemoglobin ≥6.5%	14	
High blood pressure	138	2.6
Obesity (waist circumference)	319	25.3
Body-mass index	24.9 (5.4)†	58.5

*The measured outcomes were defined as follows: Hemoglobin A1c assessment was done at the time of blood collection using point-of-care Siemens Vantage A1c testing kits (Siemens Medical Solutions USA, Malvern, PA). Blood pressure was measured in a seated position using automated sphygmomanometers (Omron HEM 705 LP, Omron Healthcare, Inc., Bannockburn, IL), with elevated blood pressure defined as systolic blood pressure ≥140 mm Hg or diastolic blood pressure ≥90 mm Hg. Waist circumference thresholds for defining obesity were ≥102cm for men and ≥88cm for non-pregnant women.

†These values represent the mean (standard deviation).

ACEs were common among study participants: the average number of ACEs was 4.9 out of a possible 16 (**Table 2**). Living with an adult who used alcohol/drugs was the most commonly reported experience (60%), followed by experiencing verbal abuse or humiliation (54%), experiencing physical abuse (51%), having a family member incarcerated (38%), and fear of being harmed (38%).

**Table 2:** Frequency of Adverse Childhood Experiences (ACEs)*

Adverse childhood experiences (n = 545)	Frequency	%
Lived with an alcohol/drug abuser	329	0.60
Verbal abuse or humiliation	292	0.54
Pushed, grabbed, slapped, or hit by an object	277	0.51
Incarcerated household family member	207	0.38
Afraid of being harmed	205	0.38
Pushed, grabbed, slapped, or hit by an object by mother	178	0.33
Lived with an adult with a mental illness	156	0.29
Kicked, bit, or punched mother	154	0.28
Parental separation or divorce	146	0.27
Scarring from physical abuse	137	0.25
Went an entire day without food	136	0.25
Went to bed hungry	122	0.22
Threatened mother with weapon	116	0.21
Went to bed thirsty	85	0.16
Touched sexually	71	0.13
Attempted/forced sex	55	0.10

*Each of the 16 adverse childhood experiences was elicited using a single questionnaire item that enquired about possible exposure to these experiences during the participant's first 18 y of life. For some items, participants were probed about the frequency of the exposure. For the purposes of analysis, all items were converted into binary indicators, with 'any' exposure categorized as 1 and 'no' exposure categorized as 0.

The unadjusted associations between cumulative number of ACEs and adult cardiometabolic risk factors and health outcomes ranged between odds ratios (OR) of 0.87 (for A1c) and 1.06 (for history of heart attack/heart failure). **Table 3** and **Table4** show the estimated associations, using multivariable logistic regression models, between the adult CVD outcomes and ACEs, while **Table 5** shows the measured adult CVD outcomes. The cumulative number of ACEs were associated with having a history of heart attack and/or heart failure (adjusted odds ratio [AOR] = 1.11, 95% confidence interval [CI], 0.999-1.234, *P* = 0.051), but the estimated association was not statistically significant. In the other regression models, the cumulative number of ACEs did not have statistically significant associations with any measures of adult CVD.

**Table 3:** Logistic regression models estimating associations between number of ACEs and adult CVD outcomes (diabetes, elevated hemoglobin A1c, heart attack/failure)

	Adult CVD outcomes (n = 545)		
	**Self-reported history of diabetes**	**Elevated hemoglobin A1c (≥6.5%)**	**Self-reported history of heart attack/heart failure**
	**Adjusted OR (95% CI)**	**Adjusted OR (95% CI)**	**Adjusted OR (95% CI)**
**Total No. ACEs**	0.95 (0.79-1.15)	0.92 (0.76-1.12)	1.11 (1.00-1.23)
**Female**	0.48 (0.14-1.64)	0.61 (0.17-2.25)	3.47 (1.21-9.99)‡
**Age (years)**	1.06 (1.02-1.10)†	1.05 (1.00-1.09)‡	1.03 (1.00-1.05)
**Education**	1.00 (0.74-1.36)	1.29 (0.98-1.70)	1.02 (0.82-1.28)
**Partnered**	1.38 (0.35-5.40)	0.94 (0.25-3.56)	0.75 (0.33-1.72)
**Assets**	1.15 (0.90-1.47)	0.92 (0.70-1.21)	1.03 (0.83-1.27)
**Body mass index**	1.05 (0.96-1.15)	1.15 (1.06-1.26)†	1.01 (0.94-1.09)

ACEs – adverse childhood experiences, CI – confidence interval, CVD – cardiovascular disease, OR – odds ratio

*Note: Each column represents the estimated regression coefficients from a single multivariable logistic regression model specifying the column header variable as the outcome and the row variables as the covariates. ‘Total No. ACEs’ represents the total number of adverse childhood experiences out of a maximum of 16. ‘Assets’ indicates the asset index score derived from a principal components analysis applied to an asset inventory of 19 total different household assets and household characteristics.

†*P* < 0.01.

‡*P* < 0.05.

**Table 4:** Logistic regression models estimating associations between number of ACEs and adult CVD outcomes (elevated cholesterol, stroke, hypertension)*

	Adult CVD outcomes (n = 545)		
	**Self-reported history of elevated cholesterol**	**Self-reported history of stroke**	**Self-reported history of hypertension**
	**Adjusted OR (95% CI)**	**Adjusted OR (95% CI)**	**Adjusted OR (95% CI)**
**Total number of ACEs**	1.10 (0.92-1.31)	1.02 (0.85-1.21)	0.96 (0.91-1.02)
**Female**	1.06 (0.20-5.78)	1.58 (0.35-7.06)	0.75 (0.49-1.20)
**Age (years)**	1.05 (1.01-1.10)‡	1.01 (0.97-1.06)	1.05 (1.03-1.06)†
**Education**	1.40 (1.01-1.93)‡	1.07 (0.75-1.51)	0.89 (0.79-1.00)‡
**Partnered**	0.86 (0.22-3.38)	1.26 (0.75-1.51)	0.89 (0.56-1.41)
**Assets**	0.66 (0.41-1.05)	0.81 (0.48-1.36)	1.08 (0.96-1.22)
**Body mass index**	1.28 (1.16-1.42)†	1.03 (0.91-1.17)	1.03 (0.99-1.08)

ACEs – adverse childhood experiences, CI – confidence interval, CVD – cardiovascular disease, OR – odds ratio

*Note: Each column represents the estimated regression coefficients from a single multivariable logistic regression model specifying the column header variable as the outcome and the row variables as the covariates. ‘Total No. ACEs’ represents the total number of adverse childhood experiences out of a maximum of 16. ‘Assets’ indicates the asset index score derived from a principal components analysis applied to an asset inventory of 19 total different household assets and household characteristics.

†*P* < 0.001.

‡*P* < 0.05.

**Table 5:** Logistic regression models estimating associations between number of ACEs and adult CVD outcomes (elevated blood pressure, obesity by waist circumference)*

	Adult CVD outcomes (n = 545)	
	**Elevated blood pressure (>140/90 mm Hg)**	**Obesity by waist circumference**	
	**Adjusted OR (95% CI)**	**Adjusted OR (95% CI)**	
**Total No. ACEs**	0.95 (0.89-1.02)	0.99 (0.93-1.05)	
**Female**	0.74 (0.46-1.19)	5.07 (3.05-8.43)†	
**Age (years)**	1.05 (1.03-1.06)†	1.02 (1.00-1.04)‡	
**Education**	0.93 (0.83-1.05)	0.95 (0.84-1.07)	
**Partnered**	1.20 (0.73-1.95)	1.19 (0.73-1.95)	
**Assets**	1.07 (0.94-1.21)	1.02 (0.88-1.17)	
**Body mass index**	1.03 (0.99-1.08)	1.22 (1.15-1.29)†	

ACEs – adverse childhood experiences, CI – confidence interval, CVD – cardiovascular disease, OR – odds ratio

*Note: Each column represents the estimated regression coefficients from a single multivariable logistic regression model specifying the column header variable as the outcome and the row variables as the covariates. ‘Total No. ACEs’ represents the total number of adverse childhood experiences out of a maximum of 16. ‘Assets’ indicates the asset index score derived from a principal components analysis applied to an asset inventory of 19 total different household assets and household characteristics.

†*P* < 0.001.

‡*P* < 0.05.

## DISCUSSION

In this cross-sectional, population-based study, we find a high prevalence of ACEs among adults living in Mbarara, Uganda. Contrary to our hypotheses, we estimated no statistically significant associations between ACEs and adult CVD outcomes. It is possible that the largely null estimated associations were driven by a relatively small number of events. However, we estimated null associations for the CVD outcomes with both a relatively high (eg, obesity, history of high blood pressure) and a relatively low (eg, positive A1c screen for diabetes; and history of elevated cholesterol, or stroke) number of events. Further, the CVD outcome that was most strongly associated with ACEs (history of heart attack/heart failure) had a relatively low number of events. Thus we believe it is unlikely that our findings are driven solely by the relatively small number of events.

The null association between ACEs and adult CVD outcomes is inconsistent with the broader literature, which suggests that adults with worse histories of childhood trauma face the greatest risk of developing a variety of CVD conditions [34,35]. Although the majority of these findings are based on data collected in high-income countries, there is not enough evidence from studies in low- and middle-income contexts to make robust conclusions about the extent to which the long-term effects of ACEs on adult CVD can be generalized across these settings. Findings from the nascent literature from low- and middle-income countries are mixed. For example, one large study of Mexican women found that ACEs significantly predicted a suite of CVD risk factors, such as hypertension, diabetes, and high cholesterol, during adulthood [7]. Conversely, another study of adults in metropolitan Manila, Philippines reported null associations between ACEs and adult CVD conditions like diabetes and stroke, but significant associations between ACEs and future risk for hypertension and ischemic heart disease [36]. The limited number of studies that report null associations between ACEs and adult cardiometabolic risk factors and cardiovascular disease outcomes may also be driven by publication bias, with those reporting statistically significant associations more likely to be published.

A number of reasons may explain the null relationships between ACEs and adverse cardiometabolic and cardiovascular outcomes in this sample. First, our models may have excluded the role of protective factors that may buffer adults from the long-term cardiometabolic and cardiovascular effects of early trauma exposure, such as the positive effects of psychological resources [37], social support [38] and cultural integration [39]. Second, the psychological and physiological impacts of certain forms of childhood trauma do not necessarily have a deterministic effect on future elevated disease risk or adversely affect the stress physiological pathways understood to underlie these long-term effects. For example, previously published studies have found null associations between early life stress and adult stress physiology outcomes, including autonomic regulation [40,41] and hypothalamic-pituitary-adrenal axis function [41,42]. Given the increased prevalence and burden of both ACEs and CVDs in low- and middle-income contexts, further research studies from these settings are needed to elucidate the possible long-term effects of ACEs on adult CVD risk.

While our study design is characterized by many strengths, including the fairly comprehensive ACEs assessment, extensive sampling of an understudied setting (relative to the rest of the ACEs and global cardiovascular health literature), and comprehensive screening of various cardiometabolic and cardiovascular health outcomes, this analysis includes a number of limitations. First, retrospective reports of ACEs may be limited by certain cognitive biases (eg, recency effect, availability heuristic), memory loss, and/or current emotional states [43-45]. Self-reported history of CVD outcomes may be similarly limited. However, correlated errors in self-report (of both ACEs and CVD outcomes) would be expected to bias our estimated associations away from the null rather than toward the null. For self-report measurement errors to bias our estimated associations toward the null, the cognitive biases, memory loss, and/or current emotional states would need to bias the exposure away from the null and bias the outcome toward the null (or vice versa). Second, the adaptation of the international ACEs survey tool may have excluded common and contextually relevant forms of child adversity, leading to a possible underreporting of childhood trauma and potential source of measurement error [46]. Finally, we did not directly assess CVD markers with the exception of hemoglobin A1c, waist circumference, and blood pressure. The use of self-reported history for most of our outcome measures may conceal possible long-term adult cardiovascular effects of ACEs due to the lack of resolution in our categorical outcome measures.

Nevertheless, this study contributes to the nascent literature on the long-term, adult cardiovascular effects of adverse childhood experiences in low- and middle-income contexts. We find that adverse childhood experiences are not associated with a suite of adult cardiometabolic risk factors and health outcomes in this sample of rural Ugandan adults. Further research in this sample is necessary to identify the pathways that may motivate the null relationship and possibly protect against adverse cardiovascular health outcomes.
